# Towards systematic evaluation of epidemic responses during humanitarian crises: a scoping review of existing public health evaluation frameworks

**DOI:** 10.1136/bmjgh-2019-002109

**Published:** 2020-01-30

**Authors:** Abdihamid Warsame, Karl Blanchet, Francesco Checchi

**Affiliations:** 1 Department of Infectious Disease Epidemiology, London School of Hygiene and Tropical Medicine, London, UK; 2 Faculty of Public Health and Policy, London School of Hygiene and Tropical Medicine, London, UK; 3 Faculty of Epidemiology and Public Health, London School of Hygiene and Tropical Medicine, London, UK

**Keywords:** public health, infections, diseases, disorders, injuries, control strategies, review, epidemiology

## Abstract

Epidemics continue to pose a significant public health threat to populations in low and middle-income countries. However, little is known about the appropriateness and performance of response interventions in such settings. We undertook a rapid scoping review of public health evaluation frameworks for emergency settings in order to judge their suitability for assessing epidemic response. Our search identified a large variety of frameworks. However, very few are suitable for framing the response to an epidemic, or its evaluation. We propose a generic epidemic framework that draws on elements of existing frameworks. We believe that this framework may potentially be of use in closing the gap between increasing global epidemic risk and the ability to respond effectively.

**Key questions**

**What is already known?**

- Effective epidemic response continues to be hampered by a number of factors. Systematic evaluations of epidemic response are a means of improving response in ongoing and future epidemics.

**What are the new findings?**

- No comprehensive epidemic response-specific evaluation framework was identified in the literature. Aspects of existing public health evaluation frameworks can be used to construct a new epidemic response evaluation framework.

**What do the new findings imply?**

- The proposed adaptive epidemic response framework constitutes a basis on which to construct a novel evaluation approach specific to epidemics. Improved evaluations support improved response.

## Introduction

Despite progress in reducing the impact of infectious diseases, they still account for between a quarter and a third of global mortality.^1^ Epidemics of these diseases disproportionately affect those in low and middle-income countries.^2^ Populations affected by humanitarian crises are also at increased risk of epidemic-driven excess mortality and morbidity.^3^ In the past half century, 20 epidemic-prone diseases including dengue, typhoid and haemorrhagic fevers have either re-emerged or spread geographically. As the world’s population continues to grow and international travel intensifies, so does the threat of epidemics.^4^

There is concern that the global ability to respond to epidemic has not kept pace with their growing threat. The failure to initially contain the Ebola pandemic in west Africa focused attention on weak international public health systems and epidemic response capabilities.^5^ The failure to respond appropriately and at scale is not confined to epidemics of international concern, but has also been a long-standing weakness at national and subnational levels, even with regard to commonly occurring pathogens such as measles, cholera and malaria.^6^ Delayed detection and declaration, decision-making based on political and economic considerations, normalisation of epidemics as routine and poor coordination and resourcing have all been posited as contributors to poor epidemic response.^6^ However, such factors are typically identified during post-response evaluations. Therefore, there remains a need to support the actors involved in epidemic response in the real-time identification and mitigation of constraining factors that reduce the effectiveness of the response itself.

The development of an epidemic response evaluation approach should be based on a comprehensive evaluation framework, which should in turn be underpinned by a clear theory of change (ToC). The latter should map how a timely epidemic response effort can lead to decreased mortality and morbidity and ultimately better health for the population, in an ideal scenario. The proposed framework should identify both the critical steps/activities/processes in a response and the various evaluation dimensions on which these can be assessed. A ToC is important in developing an evaluation framework as it provides a clear depiction of the various pathways an intervention may take towards a set of outcomes while explicitly articulating implicit assumptions. To inform the development of a robust epidemic response evaluation framework, we defined a ToC and reviewed the characteristics of existing public health emergency frameworks for both real-time and post-response evaluations. We focused our review on public health frameworks that could potentially address the design, process, output and outcome of an epidemic response rather than those focused on impact, for which epidemiological studies are usually required, and may generate findings too late to influence in time the response. Furthermore, we excluded frameworks relating to resilience to or recovery from emergencies, as our focus was on the immediate response to an epidemic.

## Methods

### Search strategy

We undertook a scoping review of the public health evaluation literature (both peer reviewed and grey) in emergency settings. A scoping review is a type of review whose primary purpose is to map the existing literature in a field of interest in terms of the volume, nature and characteristics of the primary research.^7^ The MEDLINE, EMBASE, Global Health and Web of Science databases were searched between 2008 and 2019. The following keywords were used: ‘Public health’ OR ‘health’ OR ‘nutrition’ OR ‘WASH’ OR ‘Water sanitation’ or ‘Hygiene’ AND ‘evaluation’ OR ‘assessment’ OR ‘appraisal’ AND ‘Framework’ OR ‘structure’ OR ‘Conceptual framework’ AND ‘humanitarian’ OR ‘emergency’ OR ‘disaster’ (table 1). A search of the grey literature was undertaken using Google and Google Scholar with the same search terms with results extracted from the first 100 hits. The full database-specific search strategy can be found in the online supplementary material.

10.1136/bmjgh-2019-002109.supp1 Supplementary data

**Table 1:** Search terms

Health domains	Evaluation	Humanitarian
Public health	Assessment	Emergenc*
Health	Appraisal	Disaster*
Nutrition	Framework*	Cris*s
WASH	Structure	
Water sanitation	Conceptual framework*	
Hygiene	Program* evaluation*	
	Evaluation framework*	
	evaluation* ADJ3 method*	
	Evaluation ADJ3 model*	
	Service* ADJ2evaluation*	

As this was a scoping review to build a framework rather than systematically synthesise evidence, we omitted steps characteristic of systematic reviews including hand searching of reference lists and relevant journals, consultation with experts and bias/quality grading.

### Inclusion and exclusion criteria

We included any document published in the period 2008–2019 in the English language and focused on 2018 World Bank-classified low and middle-income countries. We considered any study design but excluded evaluations of biomedical interventions (eg, drugs or medical devices), hospital-based evaluations, opinion pieces, magazine and newspaper articles.

### Data extraction and analysis

**Figure 1:**
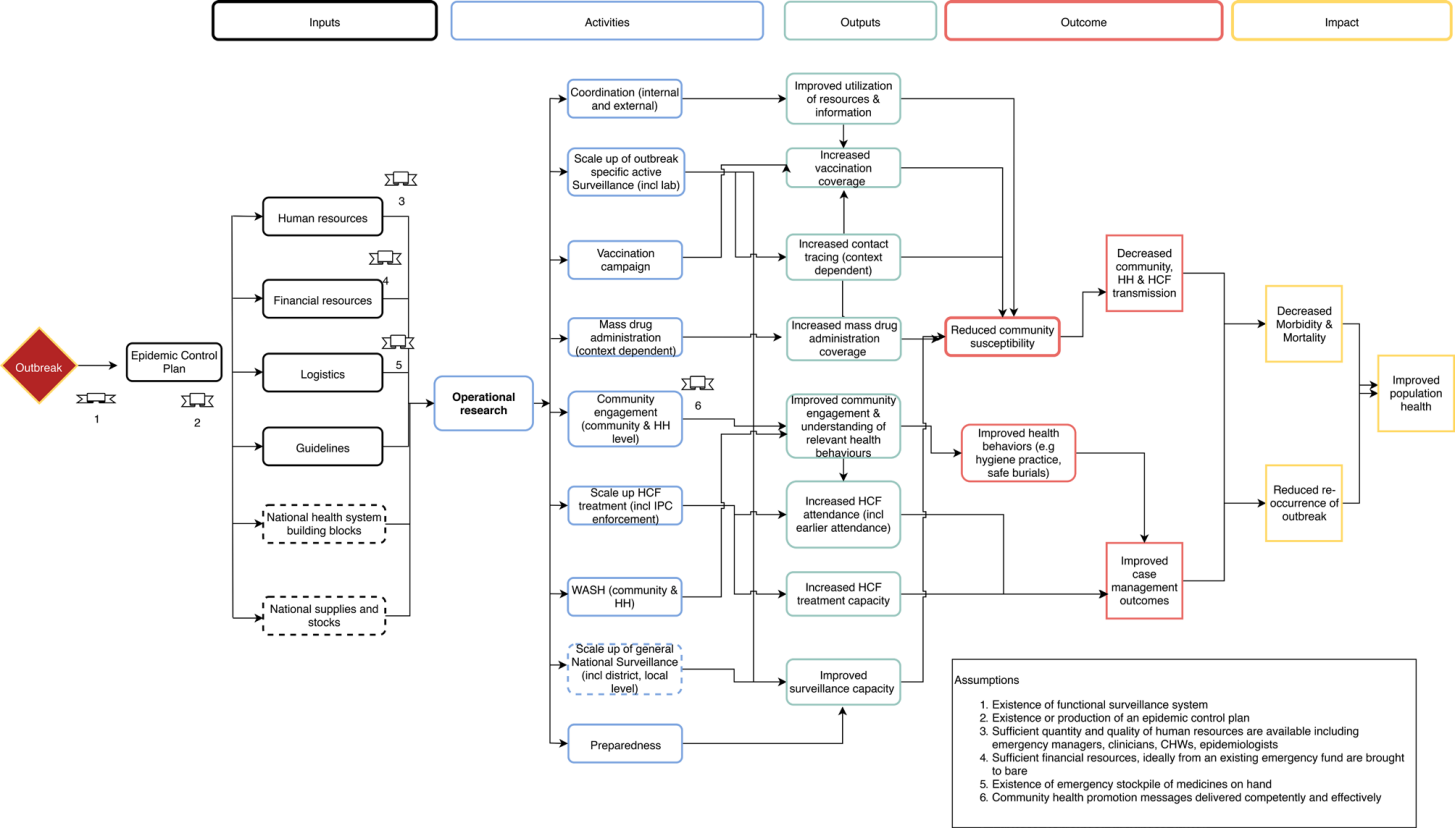
Theory of change of generic epidemic response. CHW, Community Health Worker; HCF, Health Care Facility; HH, Household; IPC, Infection Prevention & Control; WASH, Water, Sanitation and Hygiene.

We developed an epidemic response ToC for the purpose of this review as a means of identifying the various activities in an epidemic response, their linkages across the response and the potential avenues to impact (figure 1). A ToC is a model that explains how activities in an intervention can contribute to results that lead to impacts, given certain assumptions.^8^ It is useful in conceptualising programme logic and is critical for framing the monitoring and evaluation of an intervention. We used this ToC as a basis to select and assess public health evaluation frameworks identified during the literature review. Specifically, frameworks were considered for narrative synthesis when they satisfied the following criteria:

1. Can the framework be used in exploring any dimension of the ToC?
2. Does the framework encompass domains or concepts that would be useful for responders and decision-makers during an active response and/or evaluators after the response?
3. Is the framework useful for exploring the design, process, output and outcome stages of an epidemic response (ie, not focused on resilience, recovery or impact)?

In applying criterion 2, we further categorised frameworks based on their intended audience:

- Project managers and responders and primarily a guide on how to respond.
- Academics and primarily aiming to describe and deconstruct a complex situation.
- Evaluators and suggesting what to evaluate.

An extraction table listing key domains of interest was created and populated.

### Patient and public involvement

As this was a review of the literature, no patients were involved in the design of the study.

## Results

### Search output

**Figure 2:**
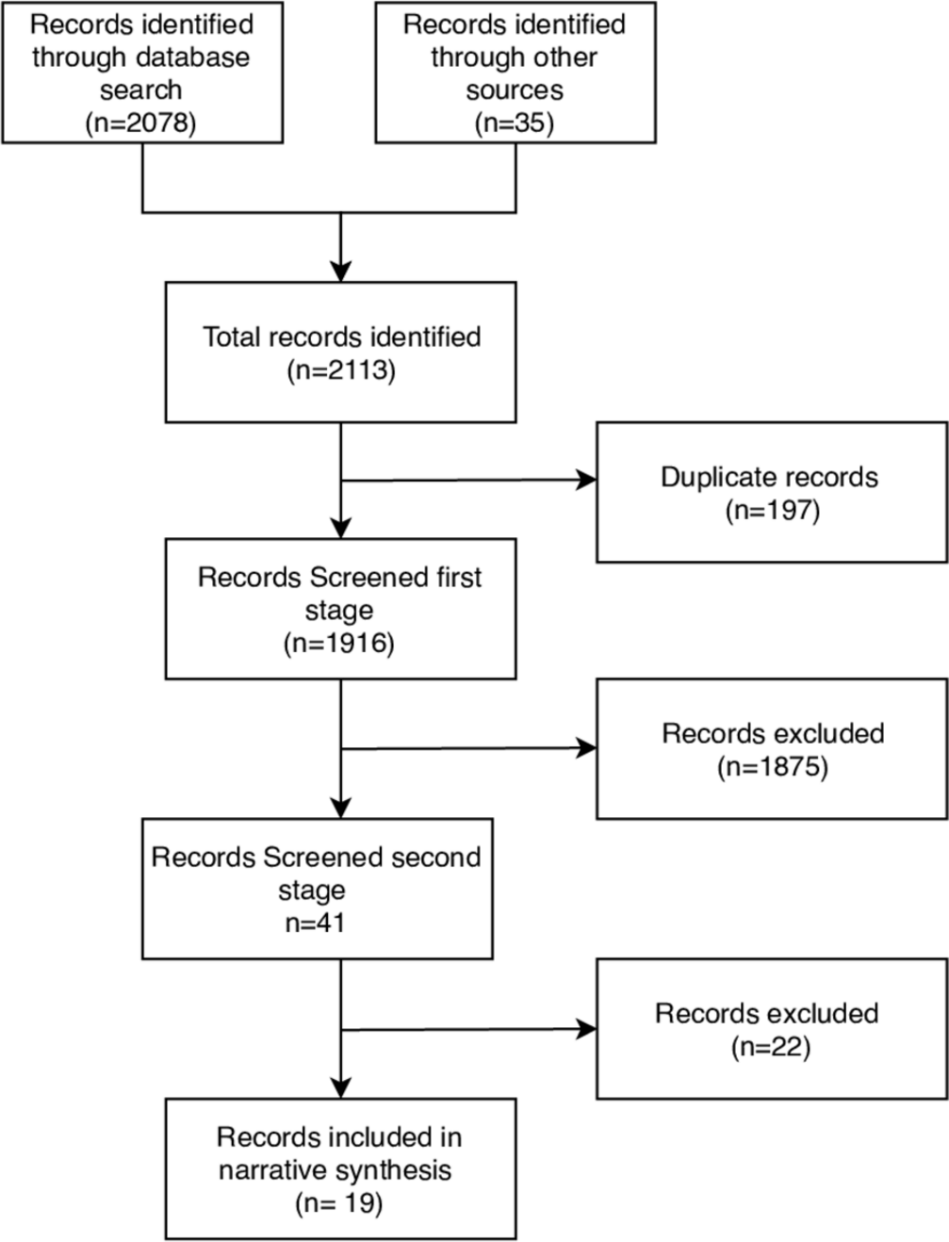
Records identified and screened in this review.

A total of 2113 records were identified (figure 2). After full-text reviews, a total of 41 documents were selected for full analysis. Among the 41 records, 39 presented or used an explicitly named framework. A further two records did not specifically name a framework but did present some evaluation criteria and dimensions that could be extracted. Of these 39 records, 1 was an epidemiological study, 6 were guidelines, 15 evaluated an intervention, 7 described an intervention but did not provide an assessment while the remainders were policy documents, guidelines or reviews of a specific health topic in emergency settings (table 2).

**Table 2:** Types of record included in the review

Type of record	Count
Epidemiological study	1
Guideline	6
Intervention study (descriptive)	7
Intervention study (evaluation)	15
Policy study	4
Review study	8
Total	41

### Key characteristics and common dimensions

The interventional studies had a wide geographical coverage with half of studies (n=12) focusing on a specific emergency-affected country or population in sub-Saharan Africa, the Middle East or South East Asia. Many of the studies also listed specific subpopulations as the targets of the intervention being studied such as internally displaced populations, refugees or victims of a natural disaster. Many of the non-interventional studies did not mention specific humanitarian contexts or populations but had a broader focus. A substantial number of records (n=16) did reference an epidemic as a type of emergency with cholera and Ebola as the most common pathogens.

Approximately one-third (n=14) of studies with explicitly named frameworks used both primary and secondary methods with roughly equal numbers using either mainly primary or secondary methods. Many studies (n=17) relied on mixed methods data for their findings while a similar number (n=16) relied mainly on qualitative data, with few (n=6) relying exclusively on quantitative data. Only half of the reports (n=19) presented indicators to be used alongside the evaluation framework: of these, 2 presented input indicators, 11 presented output indicators, 5 presented process indicators, 17 presented outcome indicators and 3 presented impact indicators.

### Description of short-listed frameworks

Table 3 lists frameworks assessed against the eligibility criteria for narrative synthesis (note that instances of similar frameworks being used across different reports have been combined into a single row).

**Table 3:** Description of frameworks derived from reports that have progressed beyond the first stage screening

Reference	Name of framework, if any	Relevant to theory of change?	Useful for responders or evaluators?	Encompasses design to outcome stages?	Progressed to narrative synthesis?
Heyse *et al* 9	Humanitarian Analysis and Intervention Design (H-AID) framework	Yes	Yes—responder focused	Yes	Yes
Wong *et al* 25	Framework for the longitudinal phases of disasters	Yes	No—academic focused	Yes—covers all stages of a response	No
Puri *et al* 29	Stages of emergency framework	No	No—academic focused	No—focused on impact	No
OECD/DAC^30^	Organisation for Economic Co-operation and Development (OECD) framework for evaluating complex emergencies	Yes—can be used to explore how response outputs are performing	Yes—evaluator focused	Yes—focused on outcomes	Yes
Murphy *et al* 21	RE-AIM framework	Yes	Yes—evaluator focused	Yes—focused on implementation of activities and potential impact	Yes
Moore *et al* 23	Framework for process evaluation of complex intervention	Yes—can be used to explore if activities are implemented as intended and relationship to outcome	Yes—evaluator focused	Yes—focused on processes	Yes
Ciglene *et al* 10	Decision-making framework for vaccination in acute humanitarian emergencies	Yes—can be used in one key epidemic response activity (vaccination)	Yes—responder focused	Yes	Yes
Altay and Labonte^11^	Integrated complexity-information flow impediment framework	Yes—information generation and flow (surveillance)	Yes—responder/decision-makers focused	Yes—process and outcome of information	Yes
Huicho *et al* 31	Framework for measuring efforts to increase access to health workers in underserved areas	No	Yes—evaluator focused	Yes—covers from design to impact	No
Oppenheim *et al* 32	Epidemic Preparedness Index framework	Yes—response activities	No—academic focused	No—preparedness focused	No
Burnham *et al*; Dobai and Tallada; Fogden *et al*; IFRC; Lam and Ly; Thormar; Darcy *et al* 14–18 33–35	IFRC and UNICEF frameworks	Yes	Yes—evaluator focused	Yes—covers all stages of a response	Yes
Nickerson *et al* 36	Health systems framework	Yes—can be used to explore input and impact of epidemic response	No—academic focused	Yes	No
Fitter *et al* 37	CDC’s Essential Package of Health Services framework for Haiti	Yes—can be used to explore how research underlays response	Yes—evaluator/academic focused	No—focused primarily in resilience	No
Heitzinger *et al* 38	Unnamed framework	Yes—evidence-based decision-making	Yes—responder focused	Yes—process	Yes
Jordans *et al* 39	Care utilisation model	No	No—academic focused	Yes—focused feasibility in design and implementation of package of service	No
Chung and Chung^40^	CBR framework	No	Yes—evaluator focus	No—focused on impact	No
Checchi *et al* 41 42	Conceptual framework of public health information domains in crises	Yes—can be used to understand chain of causality that affects epidemics	No—academic focused	No—focused on impact of drivers on mortality	No
Seeger *et al* 19	Emergency risk communication (ERC) conceptual model	Yes—can be used to explore community outreach	Yes—evaluator focused	Yes—focused on outcomes of ERC and processes	Yes
Khan *et al* 43	Resilience framework for public health emergency preparedness	No	No—academic focused	No—resilience focused	No
Campbell *et al* 44	Framework for assessment of the role of the global strategy in supporting the joining of organisations in Myanmar	No	No—academic focused	No	No
Tumilowicz *et al* 45	Implementation research framework	No	No—academic focused	Yes—process of implementation	No
Kapiriri and Be LaRose^46^	Kapiriri and Martin’s priority setting evaluation framework	Yes—prioritisation of interventions and of diseases to respond to	Yes—responder/decision-making focused	Yes—process of prioritisation	Yes
Figueroa^47^	Ideation model and pathways framework	No	No—academic focused	No	No
Desie and Ismail^48^	Accountability to Affected Population (AAP)	Yes—can be used to explore community outreach intervention	No—academic focused	Yes—used in process	No
Task Force on Quality Control of Disaster Management^49^	Longitudinal framework	No	No—academic focused	No	No
VM *et al* 50	Predictive evaluation framework	No	No—academic focused	No	No
de Jong *et al* 51	de Jong’s public health prioritisation framework	Yes—can be used to explore prioritisation of alternative epidemic control interventions	Yes—responder/academic focused	Yes—focused on programme design	Yes
Abramson *et al* 52	Resilience activation framework	No	No—academic focused	No—resilience focused	No
Savoia *et al* 20	Risk Communications Evaluation (RICE) framework	Yes—can be used to explore community outreach intervention	No—academic/evaluator focused	Yes	Yes
Sambala *et al* 53	Standardised checklist	Yes—can be adapted to explore activities and process in ongoing epidemic	Yes—responder focused	No—preparedness focused	No
Lin *et al* 54	Unnamed framework	Yes—can be used to explore the structure of the response	Yes—evaluator	Yes—impact	Yes
Van Beurden *et al* 55	Cynefin framework	No	No—academic focused	No	No
D’Ostie-Racine *et al* 56	Wholey’s (2004) framework	No	No—academic focused	No	No

CBR, community-based rehabilitation; CDC, US Centers for Disease Control and Prevention; IFRC, International Federation of Red Cross and Red Crescent Societies; RE-AIM, Reach, Efficacy, Adoption, Implementation and Maintenance.

After applying the vetting criteria in table 3, a total of 13 frameworks from 19 records were brought forward for narrative synthesis. The results show that there are a wide variety and range of frameworks for public health programmes in emergencies. These range from generic conceptual frameworks for framing an approach to disaster response to very detailed, prescriptive frameworks for evaluating specific programmes. A short description of each of the frameworks is included below grouped according to the primary target audience.

#### Responder-focused frameworks

##### Humanitarian Analysis and Intervention Design framework

This model by Heyse *et al* 9 was developed to support humanitarians in rapid, evidence-based programming in humanitarian response. It purports to do this by building understanding of the problem, possible interventions and, finally, appropriate, feasible and safe interventions given the context. The authors describe this framework as a meta-model as it draws on and synthesises elements of existing analytical and humanitarian diagnostics frameworks. The authors propose specific tools for analysing each of the three core elements: crisis contexts, interventions and stakeholders. The underlying logic of the framework is that practicable and appropriate humanitarian interventions can only be identified by linking proper contextual understanding with potential interventions and stakeholder analysis.

##### Decision-making framework for vaccination in acute humanitarian emergencies

This framework developed by the WHO and used in South Sudan^10^ provides guidance on selection of vaccination strategies in humanitarian crisis settings. It has three core components: (1) assessing the risk of vaccine-preventable disease in the local population, (2) vaccine selection and characteristics to consider, and (3) local contextual constraints that impact on timely decision-making. It is intended to be applied in both short-term and protracted crises with the outcome of saving lives and reducing the burden of disease.

##### Integrated complexity-information flow impediment framework

This framework developed by Altay and Labonte^11^ describes the complexity and resultant challenges in humanitarian information flow during the Haiti earthquake response. In it, the authors analyse the implications of these barriers on effective humanitarian response and offer recommendations on overcoming them. They propose an integrated complexity-information flow impediment framework which is an amalgamation of two concepts: complex systems and information flow impediments. Complex systems such as might be found in the inception of a humanitarian response refer to ‘the evolution of new structures and non-linear patterns arising from the inter-relationships and interconnectivity among and between elements located within a system and between that system and its environment’ while information flow impediments refer to those elements that might impede the effective flow or usage of information.

##### Kapiriri and Martin’s priority setting evaluation framework

Initially developed to identify successful priority setting in low and middle-income countries, Kapiriri and Martin’s framework was applied to priority setting with regard to tackling several disease epidemics in Uganda. The framework comprised five dimensions: (1) priority setting context, (2) prerequisites (elements, such as resource allocation, necessary for successful priority setting), (3) priority setting process (processes such as stakeholder consultation that need to be undertaken), (4) implementation, and (5) outcome and impact. The framework also provides means of verification and indicators for each of the dimensions. The framework was able to identify successful drivers of epidemic priority setting in the Ugandan context including reliable evidence collection, stable sociopolitical context and credible institutions. It also provided recommendations on areas in need of strengthening in order to better drive successful prioritisation and control of epidemics.

##### de Jong’s public health prioritisation framework

Proposed in the context of addressing the mental health burden of youth in humanitarian settings, the framework provides a means of translating programme assessments into priority activities. It comprised 10 factors to be considered in selecting and prioritising response activities: (1) locally perceived needs and concerns; (2) prevalence and incidence; (3) severity of problems and disorders; (4) treatability and feasibility; (5) expertise, knowledge and availability of practitioners; (6) ethical applicability; (7) political acceptability (eg, in managing human rights violations); (8) cultural sensitivity; (9) programme sustainability; and (10) cost-effectiveness.

#### Evaluator-focused frameworks

##### OECD/DAC framework

The Organisation for Economic Co-operation and Development/Development Assistance Committee (OECD/DAC) framework^12^ has served as a basis for a large number of evaluations,^13^ and, though meant for development settings, has been referred to in several emergency evaluations.^14–18^ The main elements in the OECD/DAC framework include relevance (degree to which the activity is suited to the priorities and policies of the target group, recipient and donor), efficiency (the measurement of outputs relative to their inputs), effectiveness (the measurement the extent to which activities achieve their purpose), impact (including the wider effect of the programme on the lives of beneficiaries) and sustainability (the extent to which the programme or impact of the programme is likely to continue after donor funding has been withdrawn).

##### International Federation of Red Cross and Red Crescent Societies and UNICEF frameworks

Our search identified several public health programme and epidemic response evaluations done by the International Federation of Red Cross and Red Crescent Societies. Although the frameworks used were not explicitly named, they did consistently consider the same core evaluation elements and were largely analogous to those first proposed by the OECD/DAC. These include ‘relevance and appropriateness, effectiveness, efficiency and sustainability’. Some evaluations included impact, coverage and coherence, as additional distinct elements. The same set of core evaluation elements was used by the UNICEF to evaluate a response to cholera in Yemen, with the inclusion of an additional element of connectedness (the extent to which a response contributes to long-term preparedness and prevention of a future epidemic).^18^ In evaluations specific to epidemic response, both organisations mapped out relevant activities such as social mobilisation, contact tracing, case management, coordination and surveillance onto the primary evaluation elements listed above.

##### Risk Communications Evaluation frameworks

The emergency risk communication (ERC) conceptual model framework by Seeger *et al* 19 focuses on evaluating ERC in public health emergencies. It is composed of three primary stages: inputs, ERC message development and dissemination process, as well as ERC outcomes. Inputs are drawn from experience of relevant parties including US Centers for Disease Control and Prevention, partners and audiences. ERC message development and dissemination process stage includes elements which are important for assessing ERC on audiences including types of messaging, sufficiency of messaging and timeliness of messaging. The framework then illustrates how these elements interact to produce short, medium and long-term outcomes in the last stage.

The Risk Communications Evaluation framework developed by Savoia *et al* 20 also focuses on evaluating risk communication in public health emergencies. Through a systematic review of the literature, the authors identified outcomes for ERC. These include information environment-level outcomes such as message content, population-level outcomes such as information-seeking behaviours, as well as system-level outcomes such as policies and mitigation strategies. They then identified processes contributing to outcomes through use of key informant interviews. Together with macro context, mission and structural capacity, the authors presented a framework which highlights potential levels of evaluations and illustrates the complexity of ERC processes through use of feedback loops.

##### Reach, Efficacy, Adoption, Implementation and Maintenance framework

A qualitative study by Murphy *et al* 21 attempted to assess a new model of diabetes healthcare implemented by Médecins Sans Frontières in a hospital in the eastern Democractic Republic of Congo. The study sought to understand patient and provider perspectives on the new model in order to determine factors that could strengthen or impede implementation. The study used the RE-AIM framework,^22^ which observes Reach (proportion of the population affected by the programme), Efficacy (negative and positive outcomes), Adoption (degree of participation), Implementation (degree to which the programme is implemented as intended) and Maintenance (institutionalisation of the programme).

##### Framework for process evaluation of complex interventions

Developed as part of the Medical Research Council's guidance on process evaluation,^23^ this framework elucidates the causal mechanisms within complex interventions that link inputs with the outcome. Complex interventions are those that contain several interacting components and are characterised by unpredictability, emergence and non-linear outcomes. Emergence refers to the appearance of complex patterns from relatively simple interactions while non-linear outcomes refer to causal steps in an outcome that are more complex than a single linear chain and include, for example, feedback loops. The importance of undertaking process evaluations is premised on the need to capture how implementation occurred in practice in order to avoid type 3 error (dismissing sound implementation theory due to a failure to implement an intervention appropriately).

##### Unnamed frameworks

Two unnamed frameworks made it to the synthesis stage of this scoping review. They include a framework developed by Lin *et al* conceptualising the response to the 2008 Sichuan earthquake. The framework is based on four domains of emergency response: leadership, medical response, public health response and societal response with each domain in turn comprised of subdomains consisting of relevant response activities. Additionally, an unnamed framework used by Heitzinger *et al* was presented as a means of assessing the success of operational research in the midst of an epidemic. Used in the 2017 Madagascar plague response, it puts forward four outcome dimensions: dissemination of results, peer-reviewed publication, changes to policy and practice, and improvements in programme performance and health.

## Discussion

In the past three decades there has been an significant surge in the production of evaluations in emergency settings.^24^ However, as previous studies have noted and our results have confirmed, there remains a wide variability in these evaluations in scope, content and audience.^25 26^ Due to time and resource constraints, our review focused on more recent frameworks in the published literature and may have missed earlier possibility relevant frameworks. Additionally, our decision to limit the search of the grey literature may also have minimised the number of relevant frameworks acquired. We have attempted to offset these limitations by intentionally opting for a broad search approach within the review time frame (2009–2018) in order to compile a wide range of frameworks from which to draw. This decision, in addition to the rapid nature of the review, provided ample variety in the frameworks compiled from both grey and peer-reviewed literature in a relatively short time frame. Nevertheless, there were some important trends that emerged. In keeping with the acknowledged importance of context in evaluation methodology,^27^ most frameworks in this review emphasised the importance of context in designing an intervention and assessing its performance. However, this often resulted in evaluation approaches that were narrowly focused on the setting in which they were used. As a result, insufficient attention was given to the potential applicability of the proposed frameworks in alternative settings and circumstances. Evaluations are cyclical and recurring process meant to assess and improve intervention performance in a stepwise fashion.^28^ Few studies in this review however explicitly mentioned the need for an iterative approach to applying their proposed frameworks but rather presented the application of the framework as a single event. Few frameworks provided any information on redesigning an intervention particularly in the event of failure to achieve outcomes or in light of unintended consequences. This is a particularly large gap given the complex and fluid nature of public health provisioning in emergency settings.^11^

Lastly, frameworks captured as part of this scoping review tended to focus on a narrow segment of an intervention’s lifespan. Many frameworks, for example, focused on the design of suitable interventions or on priority setting within the implementation phase or in many cases some aspect of performance. No framework provided a holistic all-encompassing approach to evaluating all phases of an intervention’s life cycle. Without such a framework, it is difficult to make an overall judgement of an emergency public health intervention.

With respect to epidemic response evaluation, no single overarching framework was found. Although no single framework in this review captures all potentially relevant dimensions and approaches for evaluating the response to epidemics, taken together our review provides ample material from which to construct an epidemic-specific one.

**Figure 3:**
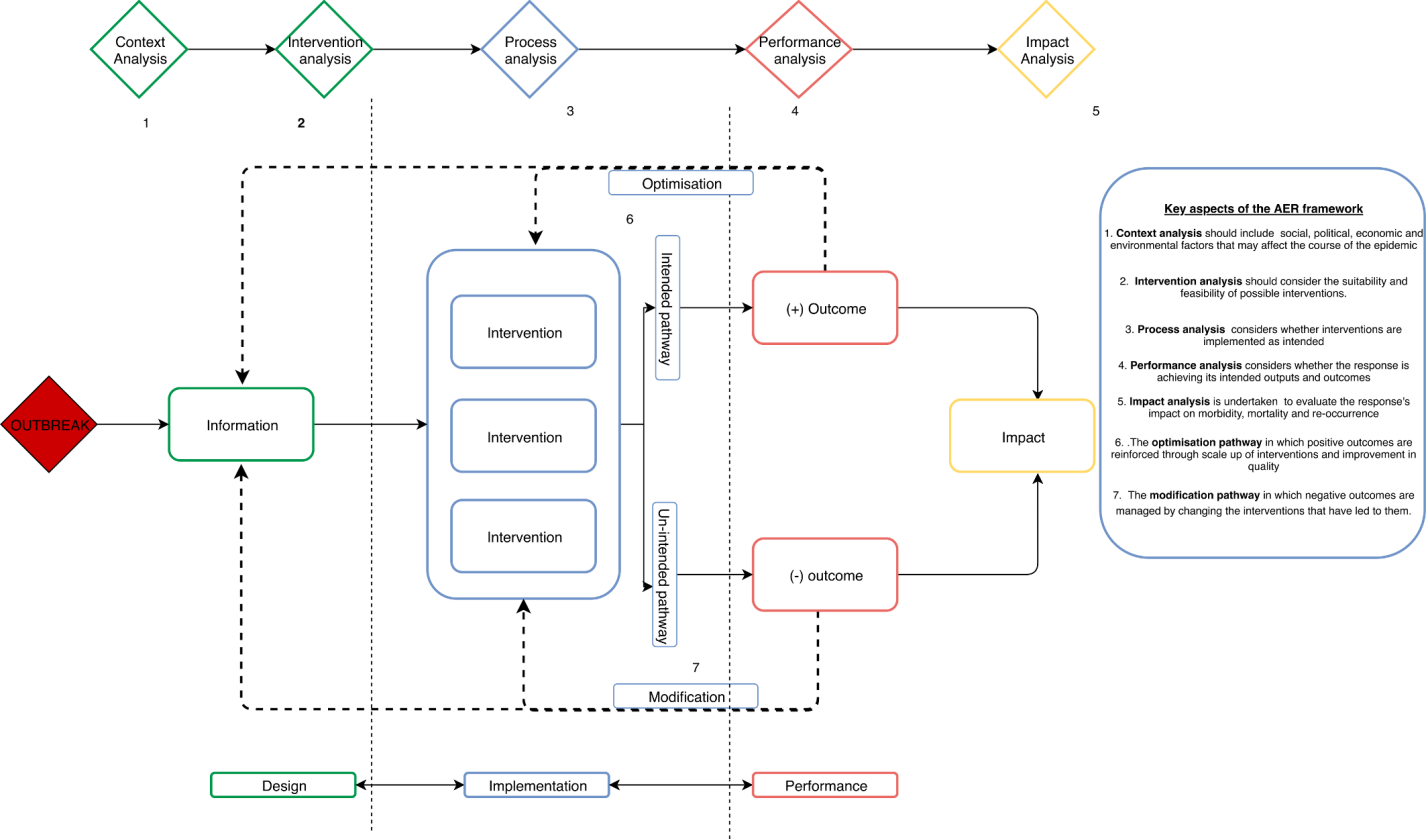
The adaptive epidemic response (AER) framework. AER, adaptive epidemic response.

We therefore propose the adaptive epidemic response (AER) framework as a means of filling this gap (figure 3). The AER framework presents key elements and activities that are primarily relevant to responders and decision-makers in the midst of an epidemic but may also be used to guide postresponse evaluations.

It is divided both vertically and horizontally. Vertically it comprised of the three interlinked components of a response: design, implementation and performance. These components do not occur linearly but are iterative and their cyclical nature is represented through double-headed arrows. Horizontally, the top of the framework presents the thread of analyses that can and should be conducted and for which specific instruments may be developed. The bottom of the framework illustrates the flow of information and activities.

As adapted from the Humanitarian Analysis and Intervention Design, the AER framework suggests that at the outset of an epidemic (1), a context analysis should be undertaken to gain understanding of social, political, economic and environmental factors that may affect the course of the epidemic. The next step (2) is to undertake an intervention analysis in which suitable and feasible interventions are considered and a package of interventions, collectively known as the response, is decided on. Possible interventions include establishing coordination mechanisms, surveillance, preventive measures (eg, vaccination, health promotion, Water Sanitation and Hygiene) and case management. In this stage, elements of de Jong’s public health prioritisation framework as well as Kapiriri and Martin’s priority setting evaluation framework can be used to undertake a structured intervention analysis and prioritise key interventions.

In the implementation phase, the selected interventions are rolled out. Here (3) a process analysis can be undertaken to explore whether these interventions are implemented as intended. Interventions may then follow two paths: that intended by responders/decision-makers and that not intended by responders. The intended pathway leads to positive outcomes such as reduction in transmission, improved health behaviour and improved case management while the unintended pathway leads to negative outcomes such as increased community hostility, increased resistance to contact tracing and as a result increased transmission.

At this stage, a performance analysis (4) may be undertaken using both quantitative and qualitative methods to describe the extent to which the response is achieving its intended outputs or outcomes, and understand reasons for the measured performance. Here the evaluator-focused frameworks can be drawn from to develop specific performance assessment instruments. Lastly, an impact analysis (5) can be done to explore impact on morbidity, mortality and reoccurrence. Both negative and positive outcomes generate information which can then be used to adapt or optimise the response. This portion of the framework (adopted from the WHO decision-making framework for vaccines in emergencies as well as framework for operational research effectiveness) is illustrated by adaptation feedback pathways going back to the design and implementation dimensions. In the case of interventions leading to positive outcomes, the response is optimised (6) through actions such as increasing the geographic accessibility of selected interventions and improving quality. In the case of interventions leading to unintended negative outcomes, the response is modified (7) through actions such as selection of different sets of interventions and/or other adjustments to the response (eg, improving coordination, better engagement with beneficiaries, and so on). The proposed framework is intended to support responders and decision-makers during an epidemic, as well as evaluators. It is meant to be sufficiently generic to be adapted to different pathogens, country settings and stages of an epidemic. Both quantitative and qualitative approaches can be used in exploring its facets in order to provide diverse but ultimately complementary information.

In order to build on the findings of this study, we intend to further refine the proposed framework through a follow-up systematic review of published epidemic response evaluations. The broad and wide approach used in this scoping review will be complimented by the depth and focused approach from the planned systematic review. Furthermore, we propose that the resulting framework be used as a starting point to develop specific analysis instruments. Lastly, we recommend that the framework and resultant analysis instruments be piloted in a variety of settings to assess the response to both ongoing and concluded epidemics.
