# Supplementary material for: Towards systematic evaluation of epidemic responses during humanitarian crises: a scoping review of existing public health evaluation frameworks
Source: BMJ Glob Health. 2020 Jan 30;5(1):e002109. doi: 10.1136/bmjgh-2019-002109 (PMC7042582; doi:10.1136/bmjgh-2019-002109)
Supplement: Supplementary data [file bmjgh-2019-002109supp001.pdf]

Supplementary Material

ANNEX

Extraction Table

No.	Year	Title	Type of paper	Detail	Framework utilized or proposed	Dimensions of the framework	Type of data collection (primary or secondary)	Type of indicators of focus	Type of data collection (Quant vs Qual)	Humanitarian Setting	Humanitarian Population	Epidemic type	Epidemic Setting

Search Strategy

Medline

1. Public Health/
2. health/ or global health/ or population health/ or public health/
3. Nutrition Surveys/ or Nutrition Assessment/
4. Sanitation/ or Hygiene/
5. exp Program Evaluation/
6. Nutrition Assessment/ or Needs Assessment/ or Health Impact Assessment/
7. apprais*.mp. [mp=title, abstract, original title, name of substance word, subject heading word, floating sub-heading word, keyword heading word, organism supplementary concept word, protocol supplementary concept word, rare disease supplementary concept word, unique identifier, synonyms]
8. framework*.mp. [mp=title, abstract, original title, name of substance word, subject heading word, floating sub-heading word, keyword heading word, organism supplementary concept word, protocol supplementary concept word, rare disease supplementary concept word, unique identifier, synonyms]
9. conceptual framework*.mp. [mp=title, abstract, original title, name of substance word, subject heading word, floating sub-heading word, keyword heading word, organism supplementary concept word, protocol supplementary concept word, rare disease supplementary concept word, unique identifier, synonyms]
10. Program* evaluation*.mp. [mp=title, abstract, original title, name of substance word, subject heading word, floating sub-heading word, keyword heading word, organism supplementary concept word, protocol supplementary concept word, rare disease supplementary concept word, unique identifier, synonyms]
11. Program Evaluation/
12. evaluation framework*.mp. [mp=title, abstract, original title, name of substance word, subject heading word, floating sub-heading word, keyword heading word, organism supplementary concept word, protocol supplementary concept word, rare disease supplementary concept word, unique identifier, synonyms]
13. (evaluation* adj3 method*).mp. [mp=title, abstract, original title, name of substance word, subject heading word, floating sub-heading word, keyword heading word, organism supplementary concept word, protocol supplementary concept word, rare disease supplementary concept word, unique identifier, synonyms]
14. (evaluation adj3 model*).mp. [mp=title, abstract, original title, name of substance word, subject heading word, floating sub-heading word, keyword heading word, organism supplementary concept word, protocol supplementary concept word, rare disease supplementary concept word, unique identifier, synonyms]
15. (service* adj2 evaluation*).mp. [mp=title, abstract, original title, name of substance word, subject heading word, floating sub-heading word, keyword heading word, organism supplementary concept word, protocol supplementary concept word, rare disease supplementary concept word, unique identifier, synonyms]
16. humanitarian.mp. [mp=title, abstract, original title, name of substance word, subject heading word, floating sub-heading word, keyword heading word, organism supplementary concept word, protocol supplementary concept word, rare disease supplementary concept word, unique identifier, synonyms]
17. emergenc*.mp. [mp=title, abstract, original title, name of substance word, subject heading word, floating sub-heading word, keyword heading word, organism supplementary concept word, protocol

supplementary concept word, rare disease supplementary concept word, unique identifier, synonyms]
18. Emergencies/
19. Disaster Planning/
20. disaster*.mp. [mp=title, abstract, original title, name of substance word, subject heading word, floating sub-heading word, keyword heading word, organism supplementary concept word, protocol supplementary concept word, rare disease supplementary concept word, unique identifier, synonyms]
21. cris*s.mp. [mp=title, abstract, original title, name of substance word, subject heading word, floating sub-heading word, keyword heading word, organism supplementary concept word, protocol supplementary concept word, rare disease supplementary concept word, unique identifier, synonyms]
22. 1 or 2 or 3 or 4 or 5 or 6
23. 7 or 8 or 9 or 10 or 11 or 12 or 13 or 14 or 15
24. 16 or 17 or 18 or 19 or 20 or 21
25. 22 and 23 and 24

Embase

1. public health/
2. health/ or global health/
3. nutrition/ or nutritional assessment/ or nutritional health/
4. environmental sanitation/
5. exp program evaluation/ or exp evaluation study/ or exp health program/ or exp program appropriateness/ or exp program effectiveness/ or exp program efficacy/ or exp program feasibility/ or exp program impact/ or exp program sustainability/
6. conceptual framework/
7. conceptual framework*.mp. [mp=title, abstract, heading word, drug trade name, original title, device manufacturer, drug manufacturer, device trade name, keyword, floating subheading word, candidate term word]
8. Program* evaluation*.mp. [mp=title, abstract, heading word, drug trade name, original title, device manufacturer, drug manufacturer, device trade name, keyword, floating subheading word, candidate term word]
9. evaluation framework*.mp. [mp=title, abstract, heading word, drug trade name, original title, device manufacturer, drug manufacturer, device trade name, keyword, floating subheading word, candidate term word]
10. (evaluation adj3 model*).mp. [mp=title, abstract, heading word, drug trade name, original title, device manufacturer, drug manufacturer, device trade name, keyword, floating subheading word, candidate term word]
11. (evaluation* adj3 method).mp. [mp=title, abstract, heading word, drug trade name, original title, device manufacturer, drug manufacturer, device trade name, keyword, floating subheading word, candidate term word]
12. (service* adj2 evaluation*).mp. [mp=title, abstract, heading word, drug trade name, original title, device manufacturer, drug manufacturer, device trade name, keyword, floating subheading word, candidate term word]
13. humanitarian.mp. [mp=title, abstract, heading word, drug trade name, original title, device manufacturer, drug manufacturer, device trade name, keyword, floating subheading word, candidate term word]
14. emergenc*.mp. [mp=title, abstract, heading word, drug trade name, original title, device manufacturer, drug manufacturer, device trade name, keyword, floating subheading word, candidate term word]

15. disaster/ or mass disaster/ or natural disaster/ or relief work/
16. cris*s.mp. [mp=title, abstract, heading word, drug trade name, original title, device manufacturer, drug manufacturer, device trade name, keyword, floating subheading word, candidate term word]
17. 1 or 2 or 3 or 4
18. 5 or 6 or 7 or 8 or 9 or 10 or 11 or 12
19. 13 or 14 or 15 or 16
20. 17 and 18 and 19

Global Health

1. exp public health/
2. public health.mp. [mp=abstract, title, original title, broad terms, heading words, identifiers, cabicodes]
3. health/ or community health/ or public health/
4. nutrition/ or nutrition programmes/
5. sanitation/ or disease prevention/
6. hygiene/ or sanitation/
7. evaluation/ or program evaluation/
8. needs assessment/ or assessment/ or health impact assessment/ or nutritional assessment/
9. project appraisal/
10. Conceptual framework*.mp. [mp=abstract, title, original title, broad terms, heading words, identifiers, cabicodes]
11. Program* evaluation*.mp. [mp=abstract, title, original title, broad terms, heading words, identifiers, cabicodes]
12. evaluation framework*.mp. [mp=abstract, title, original title, broad terms, heading words, identifiers, cabicodes]
13. (evaluation* adj3 method).mp. [mp=abstract, title, original title, broad terms, heading words, identifiers, cabicodes]
14. (evaluation adj3 model*).mp. [mp=abstract, title, original title, broad terms, heading words, identifiers, cabicodes]
15. (service* adj2 evaluation*).mp. [mp=abstract, title, original title, broad terms, heading words, identifiers, cabicodes]
16. humanitarian.mp. [mp=abstract, title, original title, broad terms, heading words, identifiers, cabicodes]
17. emergenc*.mp. or emergencies/
18. disasters/ or disaster*.mp. or natural disasters/
19. cris*s.mp. or crises/
20. 1 or 2 or 3 or 4 or 5 or 6
21. 7 or 8 or 9 or 10 or 11 or 12 or 13 or 14 or 15
22. 16 or 17 or 18 or 19

LMIC expert search

1. ((developing or less* developed or under developed or underdeveloped or middle income or low* income or underserved or under served or deprived or poor*) adj (economy or economies)).ti,ab.

2. ((developing or less* developed or under developed or underdeveloped or middle income or low* income or underserved or under served or deprived or poor*) adj (countr* or nation? or population? or world)).ti,ab.
3. (low* adj (gdp or gnp or gross domestic or gross national)).ti,ab.
4. (low adj3 middle adj3 countr*).ti,ab.
5. (lmic or lmics or third world or lami countr*).ti,ab.
6. transitional countr*.ti,ab.

7. global south.ti,ab.
8. Developing Countries/
9. "africa south of the sahara"/ or africa, central/ or africa, eastern/ or africa, southern/ or africa, western/
10. ("africa south of the sahara" or sub-saharan africa or central africa or eastern africa or southern africa or western africa).ti,ab.
11. "Democratic People's Republic of Korea"/
12. (north korea or (democratic people* republic adj2 korea)).ti,ab.
13. Cambodia/
14. cambodia.ti,ab.
15. Indonesia/
16. indonesia.ti,ab.
17. Micronesia/
18. Kiribati.ti,ab.
19. Laos/
20. (laos or (lao adj1 democratic republic)).ti,ab.
21. (marshall island* or caroline island* or ellice island* or gilbert island* or johnston island* or mariana island* or micronesia or pacific island*).ti,ab.
22. Mongolia/
23. mongolia.ti,ab.
24. Myanmar/
25. (myanmar or burma).ti,ab.
26. Papua New Guinea/
27. Papua New Guinea.ti,ab.
28. Philippines/
29. Philippines.ti,ab.
30. Timor-Leste/
31. Timor-Leste.ti,ab.
32. Vanuatu/
33. Vanuatu.ti,ab.
34. Vietnam/
35. (Viet Nam or Vietnam).ti,ab.
36. American Samoa/
37. american samoa.ti,ab.
38. exp China/
39. china.ti,ab.
40. Fiji/
41. fiji.ti,ab.
42. Malaysia/
43. malaysia.ti,ab.
44. marshall islands.ti,ab.
45. nauru.ti,ab.
46. samoa/
47. "independent state of samoa"/
48. ("independent state of samoa" or (samoa not american samoa) or western samoa or navigator islands or samoan islands).ti,ab.
49. Thailand/
50. Thailand.ti,ab.
51. Tonga/
52. tonga.ti,ab.
53. Tuvalu.ti,ab.

54. Armenia/
55. Armenia.ti,ab.
56. "Georgia (Republic)"/
57. Kosovo/
58. Kosovo.ti,ab.
59. Kyrgyzstan/
60. (kyrgyzstan or kyrgyz republic or kirghizia or kirghiz).ti,ab.
61. Moldova/
62. Moldova.ti,ab.
63. Tajikistan/
64. tajikistan.ti,ab.
65. Ukraine/
66. Ukraine.ti,ab.
67. Uzbekistan/
68. Uzbekistan.ti,ab.
69. Albania/
70. Albania.ti,ab.
71. Azerbaijan/
72. Azerbaijan.ti,ab.
73. "Republic of Belarus"/
74. (belarus or byelarus or belorussia).ti,ab.
75. Bosnia-Herzegovina/
76. (bosnia or herzegovina).ti,ab.
77. Bulgaria/
78. Bulgaria.ti,ab.
79. Kazakhstan/
80. (Kazakhstan or kazakh).ti,ab.
81. "Macedonia (Republic)"/
82. Macedonia.ti,ab.
83. Montenegro/
84. Montenegro.ti,ab.
85. Romania/
86. Romania.ti,ab.
87. exp Russia/
88. USSR/
89. (Russia or Russian Federation or USSR or Union of Soviet Socialist Republics or Soviet Union).mp.

90. Serbia/
91. serbia.ti,ab.
92. Turkey/
93. turkey.ti,ab. not animal/
94. Turkmenistan/
95. Turkmenistan.ti,ab.
96. Yugoslavia/
97. yugoslavia.ti,ab.
98. Haiti/
99. Haiti.ti,ab.
100. Bolivia/
101. Bolivia.ti,ab.
102. El Salvador/
103. El Salvador.ti,ab.

104. Guatemala/
105. Guatemala.ti,ab.
106. Honduras/
107. Honduras.ti,ab.
108. Nicaragua/
109. Nicaragua.ti,ab.
110. Belize/
111. Belize.ti,ab.
112. Brazil/
113. Brazil.ti,ab.
114. Colombia/
115. Colombia.ti,ab.
116. Costa Rica/
117. Costa Rica.ti,ab.
118. Cuba/
119. Cuba.ti,ab.
120. Dominica/
121. Dominica.ti,ab.
122. Dominican Republic/
123. Dominican Republic.ti,ab.
124. Ecuador/
125. Ecuador.ti,ab.
126. Grenada/
127. Grenada.ti,ab.
128. Guyana/
129. Guyana.mp.
130. Jamaica/
131. Jamaica.ti,ab.
132. Mexico/
133. Mexico.ti,ab.
134. Paraguay/
135. Paraguay.mp.
136. Peru/
137. Peru.ti,ab.
138. Saint Lucia/
139. (St Lucia or Saint Lucia).ti,ab.
140. "Saint Vincent and the Grenadines"/
141. Grenadines.ti,ab.
142. Suriname/
143. Suriname.ti,ab.
144. Venezuela/
145. Venezuela.ti,ab.
146. Djibouti/
147. (Djibouti or French Somaliland).ti,ab.
148. Egypt/
149. Egypt.ti,ab.
150. Jordan/
151. Jordan.ti,ab.
152. Morocco/
153. Morocco.ti,ab.
154. Syria/

155. (Syria or Syrian Arab Republic).ti,ab.
156. Tunisia/
157. tunisia.mp.
158. Gaza.ti,ab.
159. Yemen/
160. Yemen.ti,ab.
161. Algeria/
162. Algeria.ti,ab.
163. Iran/
164. Iran.ti,ab.
165. Iraq/
166. Iraq.ti,ab.
167. Jordan/
168. Jordan.ti,ab.
169. Lebanon/
170. Lebanon.ti,ab.
171. Libya/
172. Libya.ti,ab.
173. Afghanistan/
174. Afghanistan.ti,ab.
175. Nepal/
176. Nepal.ti,ab.
177. Bangladesh/
178. Bangladesh.ti,ab.
179. Bhutan/
180. Bhutan.ti,ab.
181. exp India/
182. India.ti,ab.
183. Pakistan/
184. Pakistan.ti,ab.
185. Sri Lanka/
186. Sri Lanka.ti,ab.
187. Indian Ocean Islands/
188. Maldives.ti,ab.
189. Benin/
190. (Benin or Dahomey).ti,ab.
191. Burkina Faso/
192. (Burkina Faso or Burkina Fasso or Upper Volta).ti,ab.
193. Burundi/
194. Burundi.ti,ab.
195. Central African Republic/
196. (Central African Republic or Ubangi-Shari).ti,ab.
197. Chad/
198. Chad.ti,ab.
199. Comoros/
200. (Comoros or Comoro Islands or Mayotte or Iles Comores).ti,ab.
201. "Democratic Republic of the Congo"/
202. ((democratic republic adj2 congo) or belgian congo or zaire).ti,ab.
203. Eritrea/
204. Eritrea.ti,ab.
205. Ethiopia/

206. Ethiopia.ti,ab.
207. Gambia/
208. Gambia.ti,ab.
209. Guinea/
210. (Guinea not (New Guinea or Guinea Pig* or Guinea Fowl)).ti,ab.
211. Guinea-Bissau/
212. (Guinea-Bissau or Portuguese Guinea).ti,ab.
213. Liberia/
214. Liberia.ti,ab.
215. Madagascar/
216. (Madagascar or Malagasy Republic).ti,ab.
217. Malawi/
218. (Malawi or Nyasaland).ti,ab.
219. Mali/
220. Mali.ti,ab.
221. Mozambique/
222. (Mozambique or Mocambique or Portuguese East Africa).ti,ab.
223. Niger/
224. (Niger not (Aspergillus or Peptococcus or Schizothorax or Cruciferae or Gobius or Lasius or Agelastes or Melanosuchus or radish or Parastromateus or Orius or Apergillus or Parastromateus or Stomoxys)).ti,ab.
225. Rwanda/
226. (Rwanda or Ruanda).ti,ab.
227. Senegal/
228. senegal.ti,ab.
229. Sierra Leone/
230. Sierra Leone.mp.
231. Somalia/
232. Somalia.ti,ab.
233. South Sudan/
234. south sudan.ti,ab.
235. Tanzania/
236. (Tanzania or Tanganyika or Zanzibar).ti,ab.
237. Togo/
238. (Togo or Togolese Republic).ti,ab.
239. Uganda/
240. Uganda.ti,ab.
241. Zimbabwe/
242. (Zimbabwe or Rhodesia).ti,ab.
243. Angola/
244. angola.ti,ab.
245. Cameroon/
246. Cameroon.ti,ab.
247. Cape Verde/
248. (Cape Verde or Cabo Verde).ti,ab.
249. Congo/
250. (congo not ((democratic republic adj3 congo) or congo red or crimean-congo)).ti,ab.
251. Cote d'Ivoire/
252. (Cote d'Ivoire or Ivory Coast).ti,ab.
253. Ghana/
254. (Ghana or Gold Coast).ti,ab.

255. Kenya/
256. kenya.mp.
257. Lesotho/
258. (Lesotho or Basutoland).ti,ab.
259. Mauritania/
260. Mauritania.ti,ab.
261. Nigeria/
262. Nigeria.ti,ab.
263. Atlantic Islands/
264. (sao tome adj2 principe).ti,ab.
265. Sudan/
266. (Sudan not south sudan).ti,ab.
267. Swaziland/
268. Swaziland.ti,ab.
269. Zambia/
270. (Zambia or Northern Rhodesia).ti,ab.
271. Botswana/
272. (Botswana or Bechuanaland or Kalahari).ti,ab.
273. Equatorial Guinea/
274. (Equatorial Guinea or Spanish Guinea).ti,ab.
275. Gabon/
276. (Gabon or Gabonese Republic).ti,ab.
277. Mauritius/
278. (Mauritius or Agalega Islands).ti,ab.
279. Namibia/
280. Namibia.ti,ab.
281. South Africa/
282. South Africa.ti,ab.
283. or/1-282 [ALL COUNTRIES DESIGNATED AT LMIC]

Web of Science Search Terms

1) TS= (public health OR health OR nutrition OR Water sanitation OR hygiene)
Indexes=SCI-EXPANDED, SSCI, A&HCI, CPCI-S, CPCI-SSH, ESCI Timespan=All years
2,138,300
2) TS=(Conceptual framework* OR Program* evaluation* OR evaluation framework* OR evaluation* NEAR/3 method* OR evaluation NEAR/3 model* OR service* NEAR/2 evaluation*)
Indexes=SCI-EXPANDED, SSCI, A&HCI, CPCI-S, CPCI-SSH, ESCI Timespan=All years
398,173
3) TS=(Humanitarian OR emergenc* OR disaster* OR Cris*s)
Indexes=SCI-EXPANDED, SSCI, A&HCI, CPCI-S, CPCI-SSH, ESCI Timespan=All years
720,724
4) WC=(Public, Environmental & Occupational Health)
Indexes=SCI-EXPANDED, SSCI, A&HCI, CPCI-S, CPCI-SSH, ESCI Timespan=All years
912,527
5) #4 AND #3 AND #2 AND #1
Indexes=SCI-EXPANDED, SSCI, A&HCI, CPCI-S, CPCI-SSH, ESCI Timespan=All years
676

Grey Literature Search terms

GOOGLE

(public health OR health OR nutrition OR Water sanitation OR hygiene) AND ((((Conceptual framework* OR Program* evaluation* OR evaluation framework* OR evaluation* AROUND(3) method* OR evaluation AROUND(3) model* OR service* AROUND(2) evaluation*)))) AND (humanitarian OR emergenc* OR cris*s) Related: www.who.int
